# Supplementary material for: Can alcohol consumption in Germany be reduced by alcohol screening, brief intervention and referral to treatment in primary health care? Results of a simulation study
Source: PLoS One. 2021 Aug 5;16(8):e0255843. doi: 10.1371/journal.pone.0255843 (PMC8341530; doi:10.1371/journal.pone.0255843)
Supplement: Attachment — Submitted filename: Comments.docx [file pone.0255843.s003.docx]

Dr. Manthey and collaborators investigated whether the implementation of an alcohol screening, during the primary health care visit, and the brief intervention and/or referral treatment would reduce the amount of drinking levels and heavy episodic drinking in Germany population. Authors used a very robust model to simulate different scenarios (based in the percentage of patients screened). They controlled the data for sex, age, and educational levels, and also counted for the under-reported drinking levels, since the data input was acquired from different surveys. Their simulation showed that if 50% of the primary health care patients would be screened for alcohol use, the consumption of this drug could be reduced in 12%. Authors, also highlights the importance of making their model open access as a tool for other studies. In general, the study is timely, the bioinformatic tools applied are sound, the authors provide clear models and hypothesis, and the findings are clearly embedded in a theoretical framework in the discussion section.

The figures resolution needs to be improved. I could not read the diagram in figure 1. Authors could add to the discussion their perspective about why the screening is not efficient to reduce the heavy episodic drinking.
