# Supplementary material for: Can alcohol consumption in Germany be reduced by alcohol screening, brief intervention and referral to treatment in primary health care? Results of a simulation study
Source: PLoS One. 2021 Aug 5;16(8):e0255843. doi: 10.1371/journal.pone.0255843 (PMC8341530; doi:10.1371/journal.pone.0255843)
Supplement: Attachment — Submitted filename: Response to Reviewers.docx [file pone.0255843.s004.docx]

Reviewer #1:

Dr. Manthey and collaborators investigated whether the implementation of an alcohol screening, during the primary health care visit, and the brief intervention and/or referral treatment would reduce the amount of drinking levels and heavy episodic drinking in Germany population. Authors used a very robust model to simulate different scenarios (based in the percentage of patients screened). They controlled the data for sex, age, and educational levels, and also counted for the under-reported drinking levels, since the data input was acquired from different surveys. Their simulation showed that if 50% of the primary health care patients would be screened for alcohol use, the consumption of this drug could be reduced in 12%. Authors, also highlights the importance of making their model open access as a tool for other studies. In general, the study is timely, the bioinformatic tools applied are sound, the authors provide clear models and hypothesis, and the findings are clearly embedded in a theoretical framework in the discussion section.

The figures resolution needs to be improved. I could not read the diagram in figure 1. Authors could add to the discussion their perspective about why the screening is not efficient to reduce the heavy episodic drinking

***Response:***

Thank you for your positive assessment of our work. We have improved the resolution of Figure 1 to 300 DPI. Further, we have added a paragraph explaining possible reasons why we found little support for reducing heavy episodic drinking (HED):

“Why did we find little support for the impact of BI for reducing HED prevalence? According to the Cochrane review which provided the effect sizes for our simulation, only three out of ten trials found BI to reduce HED risk [24]. In fact, the only German trial on that matter also found no BI effects on HED status [44]. A number of literature reviews on that matter suggest that BI in PHC settings are most impactful among drinkers who are not dependent and not seeking treatment [45]. In this population, HED is not commonly reported [46], thus BI effects may be restricted to reduce the frequency of occasions with low to moderate amounts of alcohol intake.”

Reviewer #2:

The study is well-designed and the methodology is frankly conveyed. The authors' expertise is fully evident. The paper has evident policy implications, suggesting that other alcohol policies besides SBIRT likely are needed in order to achieve short-term reduction of alcohol use and related harms in Germany.

***Response:*** Many thanks for your positive assessment of our work.
